# Probiotics for prevention of radiation-induced diarrhea: A meta-analysis of randomized controlled trials

**DOI:** 10.1371/journal.pone.0178870

**Published:** 2017-06-02

**Authors:** Meng-Meng Liu, Shu-Ting Li, Yan Shu, He-Qin Zhan

**Affiliations:** Department of Pathology, Anhui Medical University, Hefei, Anhui, Anhui Province, China; University Hospital Llandough, UNITED KINGDOM

## Abstract

**Background:**

Radiotherapy is commonly used for abdominal or pelvic cancer, and patients receiving radiotherapy have a high risk developing to an acute radiation-induced diarrhea. Several previous studies have discussed the effect of probiotics on prevention of radiation-induced diarrhea, but the results are still inconsistent.

**Objective:**

We performed a meta-analysis of randomized controlled trials (RCTs) to evaluate the efficacy of probiotic supplementation for prevention the radiation-induced diarrhea.

**Methods:**

Relevant RCTs studies assessing the effect of probiotic supplementation on clinical outcomes compared with placebo were searched in PubMed, EMBASE, and the Cochrane Library databases (up to March 30 2016). Heterogeneity was assessed with *I*^*2*^ and *H*^*2*^, and publication bias was evaluated using sensitive analysis.

**Results:**

Six trials, a total of 917 participants (490 participants received prophylactic probiotics and 427 participants received placebo), were included in this meta-analysis. Compared with placebo, probiotics were associated with a lower incidence of radiation-induced diarrhea (RR: 0.55; 95% CI: 0.34–0.88; *P* = 0.01; *I*^*2*^: 87%; 95% CI: 75%-94%; *H*^*2*^: 2.8; 95% CI: 2.0–4.0). However, there is no significant difference in the anti-diarrheal medication use (RR: 0.68; 95% CI: 0.40–1.14; *P* = 0.14) or bristol scale on stool form (RR: 0.64; 95% CI: 0.35–1.17; *P* = 0.14).

**Conclusion:**

Probiotics may be beneficial to prevent radiation-induced diarrhea in patients who suffered from abdominal or pelvic cancers during radiotherapy period.

## Introduction

Cancers are well known the leading causes of the death. There are more than 14 million new cancer cases and 8.2 million cancer patients die of cancers each year [1,2]. Admittedly, radiotherapy either alone or combined with chemotherapy, has been proved to be an effective treatment on a number of tumors. It is now considered as the cornerstone in the treatment of cancer patients at some points in the development and progression of cancer. Despite the effectiveness of radiotherapy, the side effects including diarrhea, nausea and vomiting cannot be ignored [3]. The radiation-induced diarrhea is a commonly and potentially severe complication. The possible mechanism of radiation-induced diarrhea may due to the malabsorption of lactose and bile acids, the changes of intestinal flora and intestinal motility which lead to impaired secretion, absorption and immune function of the digestive tract [3, 4]. However, once stopping the radiotherapy, gastrointestinal symptoms are still existing, which exert a negative influence on the quality of patients’ lives [5–11]. So it is important to interpret the mechanism of radiation-induced diarrhea and to explore the potential preventive options.

Probiotics are viable nonpathogenic micro-organisms which could exert a potential positive influence on our body through keeping the balance of the microbiota [12]. To date, several studies have explored its preventive and therapeutic effects on radiation-induced diarrhea. Probiotics for prevention radiation-induced diarrhea have quickly evolved, but recently published RCTs conveyed conflicting results [3, 13–19]. In order to systematically review the evidence on the preventive effect of probiotics for radiation-induced diarrhea, we performed this meta-analysis.

## Materials and methods

### Methods

The present meta-analysis was designed according to the guidelines proposed by the Cochrane Collaboration in the Cochrane Handbook for Systematic Reviews of Interventions (http://www.cochrane-handbook.org) and reported in compliance with the PRISMA (Preferred Reporting Items for Systematic Reviews and Meta-Analyses) statement [20, 21]. There is no protocol for this meta-analysis.

### Literature search

We performed a systematic literature search of PubMed, EMBASE, and the Cochrane Library from inception to March 30, 2016. We conducted electronic searches by using Medical Subject Headings (MeSH) terms and corresponding keywords. No method or language restrictions were applied and studies from all countries were eligible. The exact search strategy is shown in Appendix 1. In addition, we also searched on Clinical Trials.gov registry (https://clinicaltrials.gov/), further screened bibliographies of included studies and relevant reviews, and hand-searched the conference abstracts for unpublished work.

### Study eligibility and selection

Two investigators (Meng-Meng Liu and Shu-Ting Li) independently executed the initial search and reviewed all identified records for inclusion using predetermined criteria. All records were screened by title or abstract for relevance, and identified as included, excluded or required further retrieval to identify eligibility. Discrepancies were resolved through discussion by the review team.

Studies were included if they met the following criteria: (1) the study population comprised patients who underwent radiotherapy; (2) the intervention group received probiotics for prevention; (3) the control group received placebo or any other base ingredients; (4) There is one or more following outcomes reported: incidence of radiation-induced diarrhea, incidence of anti-diarrhea medication use or bristol scale on stool form.

### Data review and extraction

Two independent reviewers (Meng-Meng Liu and Yan Shu) extracted details from each included trials. The extracted data contained: primary author, year of publication, country, sample size of two groups, demography, primary tumor site, type of therapy, total radiation dose, accompanied chemotherapy, probiotics (microbial strain, medication dosage, administration route, administration time, and product source). Extracted data were entered into a standardized data extraction form. We also obtained the supplementary of included trials from database or contacted authors of the original studies for additional data of the case. Disagreements were resolved by an independent adjudicator (He-Qin Zhan).

### Quality assessment

We applied the Cochrane Risk of Bias tool to assess the risk of bias without masking the trial name [20, 21]. Two reviewers (Meng-Meng Liu and He-Qin Zhan) respectively labeled (“low”, “unclear”, or “high” risk of bias) each trial on following domains: random sequence generation, allocation concealment, blinding of participants and personnel, blinding of outcome assessment, incomplete outcome data, selective reporting and other bias. If one or more key domains were judged to be at high risk for a trial, it would be considered as at high risk of bias overall. If all key domains were judged to be low risk for a trial, it would be considered as at low risk of bias, otherwise it would be considered as at unclear risk of bias [22].

### Statistical analysis

We calculated relative risks (RRs) with 95% confidence intervals (CIs) for dichotomous outcomes and mean differences (MDs) with 95% CIs for continuous outcomes (all continuous outcomes reported in identical scales across all trials). Statistical heterogeneity was assessed with the *I*^*2*^ and *H*^*2*^ statistic. *I*^*2*^ values of 25%, 50%, and 75% have been suggested as indicators of low, moderate and high heterogeneity, respectively [23, 24], *H*^*2*^*<*1.2 and *H*^*2*^>1.5 were suggested as indicators of no heterogeneity and having heterogeneity [25]. The fixed-effect model would be put into use if there was no significant heterogeneity (*P*>0.05) among these studies. Otherwise, a random-effect model was used for further analyses using inverse variance (IV) method [26]. Potential publication bias was assessed by sensitive analysis [27–29]. All statistical analysis was conducted by RevMan 5.3 and Stata 13.0 software.

## Results

### Study selection

**Fig 1:**
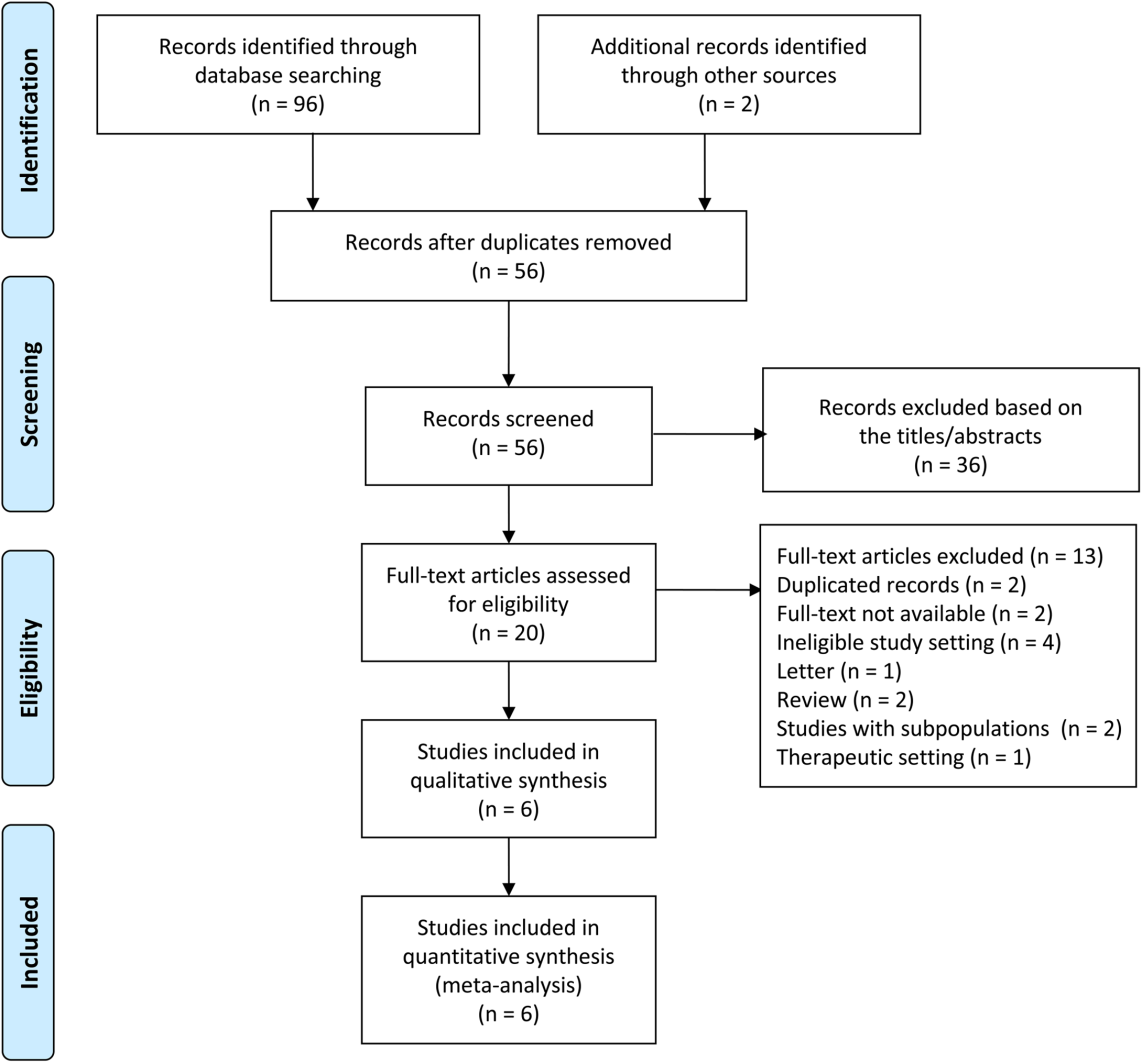
PRISMA flow diagram of the search result of the meta-analysis.

A flowchart showed the process of the selection for this meta-analysis in Fig 1. The electronic database searches and manual searches identified 98 articles. A total of 42 studies were excluded due to duplicated records, and 36 records were excluded after inspection of titles and abstracts. The remaining 20 articles were retrieved and full texts analyzed. After application of the eligibility criteria, only six RCTs were finally included in the meta-analysis [3, 13–17].

### Study characteristics

The basic characteristics of the included trials were summarized in Table 1. The year of publication of included studies ranged from 1988 to 2014. A total of 917 participants were included in this meta-analysis. There were four studies receiving *lactobacillus* (the dosage ranging from 3×10^9^ CFU to 1.35×10^12^ CFU) and two studies receiving *lactobacillus acidophilus* plus *bifidobacterium bifidum* (the dosage ranging from 2.6×10^9^ CFU to 4×10^9^ CFU). Among them, 490 participants received prophylactic probiotic supplementation and 427 participants were regarded as control group. The most of participants suffered from abdominal or gynaecological malignancies. Two trials only have applied radiotherapy, and participants of the other four trials have received radiotherapy accompanied by chemotherapy. The total radiation dosage for each cancer patient ranged from 40Gy to 80Gy. In addition, the dosages and strains of probiotics in different studies were also quite different.

**Table 1:** Characteristics of all included studies.

First author	Year/Area	Mean age	Probiotics/Placebo	Primary Tumor Site	Type of Therapy	Total Radiation Dose	Chemotherapy	Type of Probiotics	Daily Dosage	Medication usage	Route	Timing	Probiotics Source	Diarrhea grade
Salminen	1988/Finland	40–75	11/10	Cervix or uterus carcinoma	Internal and external pelvic RT and intracavitary caesium	50Gy for pelvic, 80Gy for the tumour	Intracavitary caesium	Lactobacillus	2×10^9^CFU	q.d.	Oral	5 days prior to RT,10 days after finishing RT	NA	NR
Delia	2007/Italy	No	243/239	Sigmoid, rectal or cervical cancers	Postoperative RT	60–70 Gy	Not specified	Lactobacilli, Bifidobacteria, Streptococcus	1.35×10^12^CFU	t.i.d.	Oral	The first day of RT until the end of therapy	VSL Pharmaceuticals, Fort Lauderdale, MD, USA	WHO grading
Giralt	2008/Spain	≥18	44/41	Endometrial adenocarcinoma or advanced cervical squamous cell carcinoma	Postoperative RT concomitant weekly cisplatin (only for patients with cervical cancer)	45–50.4 Gy	Weekly Cisplatin 40mg/m^2^	Streptococcus, Lactobacillus	3×10^8^CFU	t.i.d.	Oral	One week	NR	Common Toxicity Criteria of the NCI
Castro	2009/Brazil	NR	20/20	Cervical or endometrial cancer	RT treatments	NR	Not specified	Lactobacillus,	NR	NR	Oral	NR	NR	Common Toxicity Criteria of the NCI
Chitapanarux	2010/Thailand	18–65	32/31	Cervical cancer	Pelvic RT and weekly cisplatin	200 cGy per fraction, five fractions per week	Weekly cisplatin 40 mg/m^2^ for 6 weeks	Lactobacillus, Bifidobacterium	4×10^9^CFU	b.i.d	Oral	7 days before RT and continuing everyday during RT	Laboratio, Farmaceutico SIT, Mede, Italy	Common Toxicity Criteria of the NCI
Demers	2014/Canada	>18	140/86	Gynecologic, rectal, or prostate cancer	RT for gynecologic cancers without chemotherapy, gynecologic or rectal cancers with chemotherapy	40 Gy for the pelvic level	Not specified	Lactobacillus, Bifidobacterium	2.6×10^9^CFU or 3×10^10^CFU	b.i.d or t.i.d.	Oral	From the first day and ended on the last day of RT	Bifilact, Virage Santé Québec city, Canada	WHO grading

Abbreviation; CFU: colony forming units; NA: not applicable; NR: not reported; RT: radiotherapy; WHO: World Health Organization; NCI: National Cancer Institute.

### Risk of bias and quality assessment

**Fig 2:**
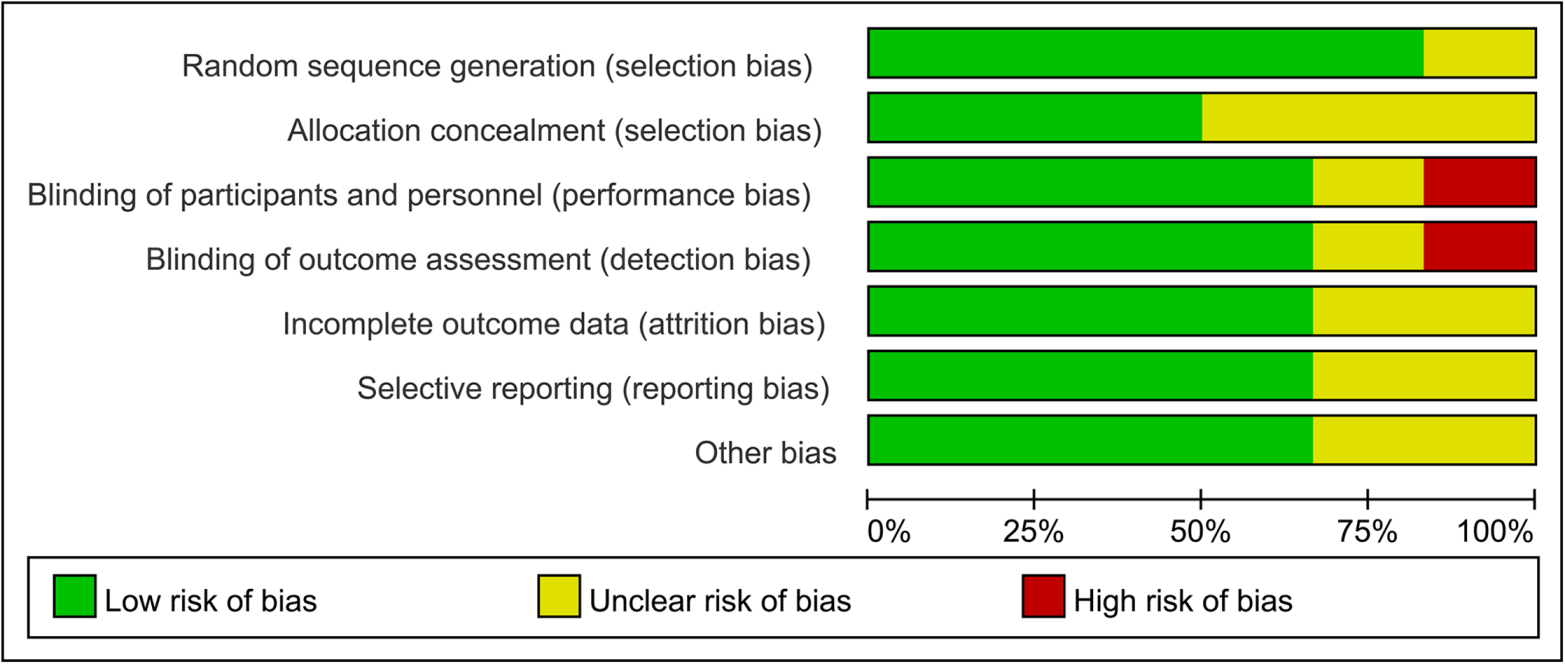
Risk of bias.

**Fig 3:**
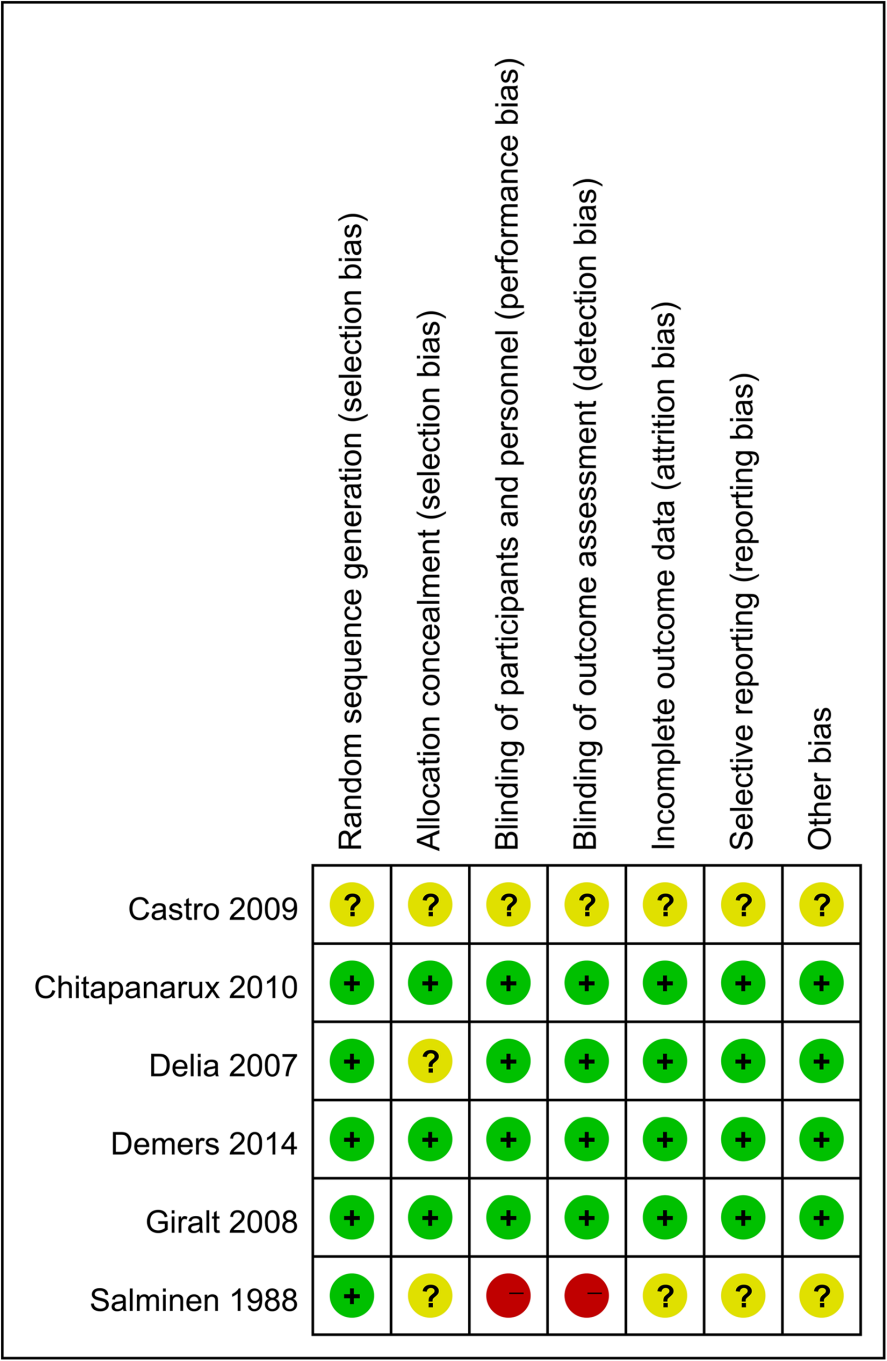
Risk of bias summary.

The overall details of risk of bias were shown in Figs 2 and 3. Three trials were categorized as being at low risk of bias, two as being at unclear risk of bias and one as being at high risk of bias. The majority of included studies reported the randomized sequence generation (5/6), while the allocation of treatment was concealed partially (3/6). In addition, four studies reported that participants and outcome assessors were blind to the intervention, and two studies reported a power calculation but none of these studies reported an intention-to-treat analysis.

### Primary outcome

**Fig 4:**
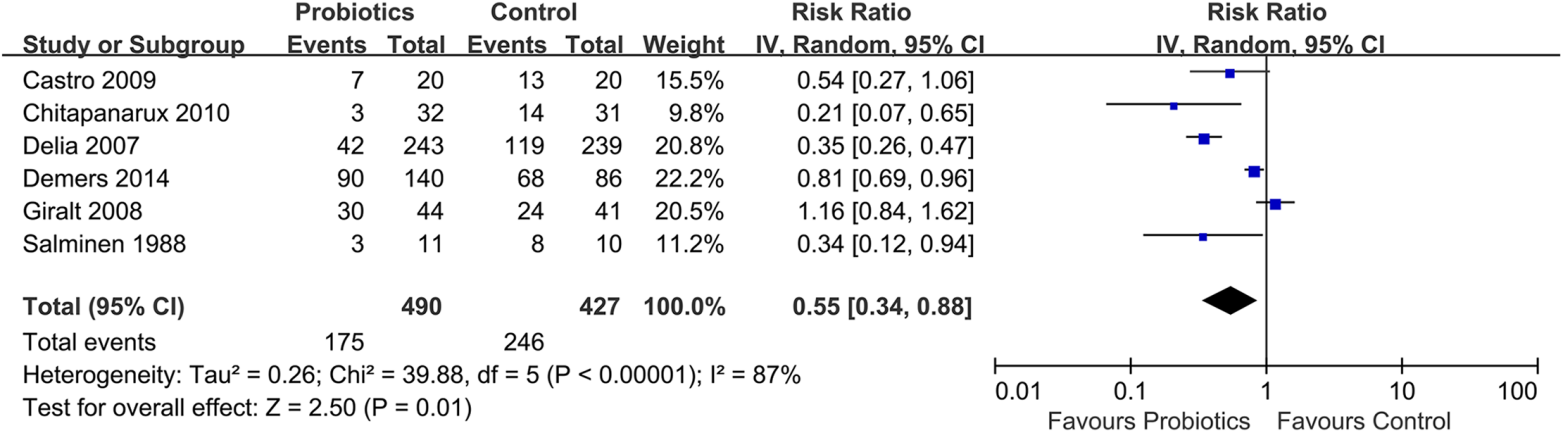
Effect of probiotics on prevention of radiation-induced diarrhea compared with placebo.

Six trails including 917 participants were provided to this meta-analysis (Fig 4). Compared with the control group, probiotic supplementation group has a significantly reduction in the incidence of radiation-induced diarrhea (RR: 0.55; 95% CI: 0.34–0.88; *P* = 0.01), with significant heterogeneity (*I*^*2*^: 87%; 95% CI: 75%-94%; *H*^*2*^: 2.8; 95% CI: 2.0–4.0).

### Secondary outcomes

**Fig 5:**
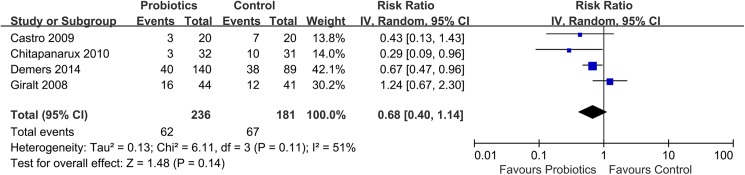
Effect of preventive probiotics on incidence of anti-diarrheal medication use compared with placebo treatment.

**Fig 6:**
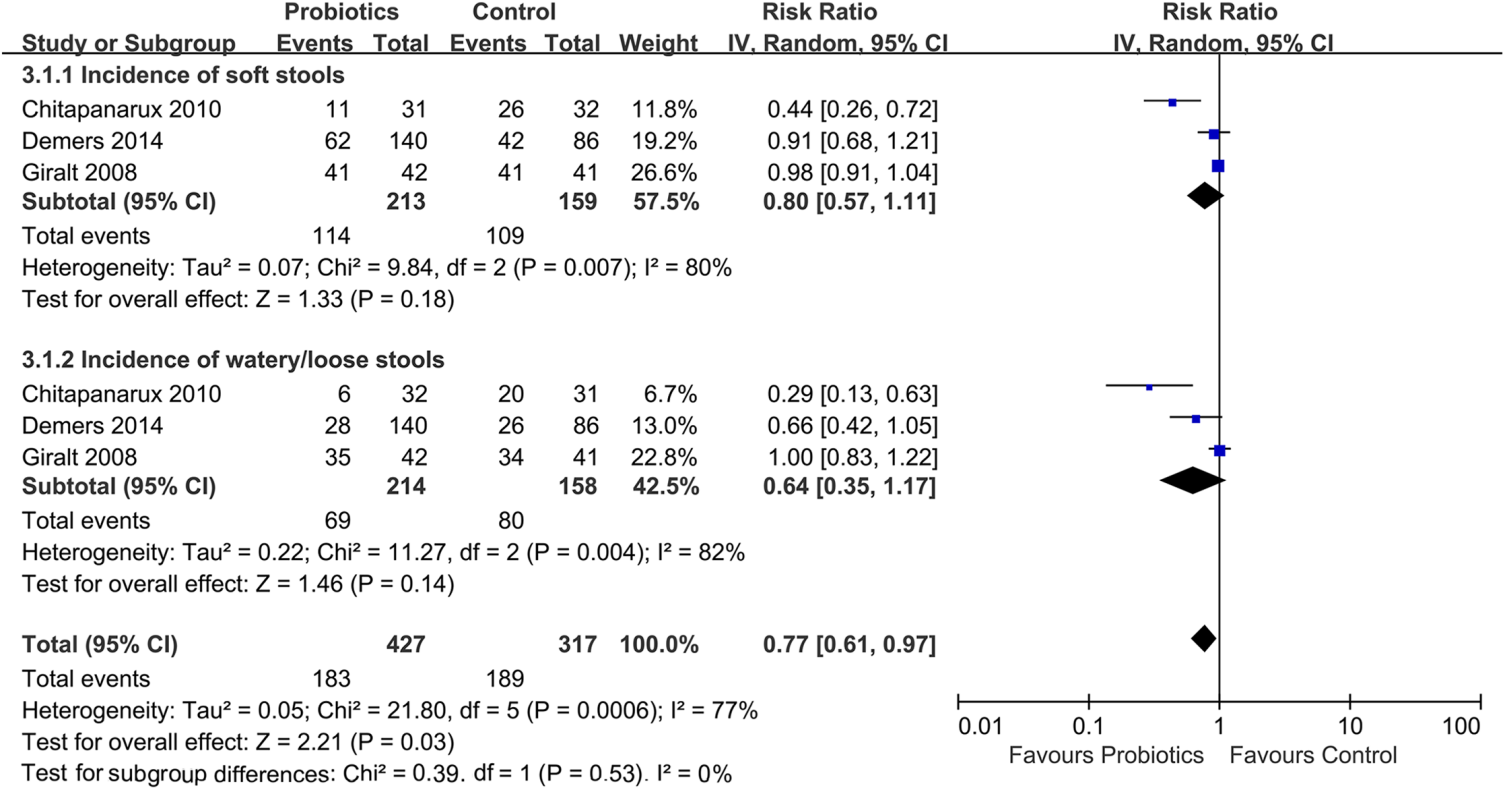
Effect of preventive probiotics on bristol scale on stool form compared with control treatment.

Compared with placebo treatment, preventive probiotic therapy has no improvement on the anti-diarrhea medication use (RR: 0.68; 95% CI: 0.40–1.14; *P* = 0.14; *I*^*2*^: 51%; 95% CI: 0%-84%; *H*^*2*^: 1.4; 95% CI: 1.0–2.5) (Fig 5) or bristol scale on stool form (RR: 0.64; 95% CI: 0.35–1.17; *P* = 0.14; *I*^*2*^: 82%; 95% CI: 45%-94%; *H*^*2*^: 2.4; 95% CI: 1.4–4.2) (Fig 6).

### Publication bias

Due to the limited number of studies having<10 studies included, the funnel plot and Egger test were not proper to evaluate the publication bias. Therefore, we performed a sensitivity analyses to assess the publication bias. After excluding one low-quality studies, we observed that the publication bias was reduced in this meta-analysis (Table 2).

**Table 2:** Publication bias in sensitivity analysis.

Meta-Analysis	No. Trials	Net change(95%CI)	*P*(*I*^2^%)*
**A**	6	0.55(0.34, 0.88)	<0.001(87.0)
***P* for bias**		0.57	
**B**	5	0.67(0.44, 1.03)	= 0.001(78.3)
***P* for bias**		0.70	

*P for heterogeneity.

## Discussion

### Main findings

Our meta-analysis comprehensively and systematically reviewed the current available literature. The result revealed that probiotic group showed the modest beneficial effect. Probiotic supplementation could be regarded as a potential adjunct therapy to appreciably relieve the clinical severity of radiation-induced diarrhea.

### Comparison with previous meta-analyses

To the best of our knowledge, until 2009, there was only one meta-analysis which consisting of 632 subjects published on using probiotics to prevent acute radiation-induced diarrhea [30]. It concluded that probiotic therapy had no beneficial effect in the prevention or treatment of radiation-induced diarrhea. In comparison, our present meta-analysis included more recent studies, which included 6 trials with 917 patients for prophylaxis, and we conveyed a different conclusion, that probiotic could effectively reduce the incidence of radiation-induced diarrhea. Due to the lack of new study focusing on the treatment efficacy of probiotics for radiation-induced diarrhea, we just performed a meta-analysis regarding to the preventive effects.

### Possible mechanisms for findings

To date, the mechanism of radiation-induced diarrhea is still unclear. The widely accepted hypotheses are as follows. Firstly, the intestine has a complex microbial ecosystem which is of utmost important and specific function [14, 17, 30–32]. However, radiation produces a burst of free radicals, which not only aims at cellular DNA, but also alters proteins, lipids, carbohydrates and complex molecules.They may cause the disruption of micro-ecology, the change of bacterial flora and the host’s homeostasis, which can result in an unregulated process of apoptosis of epithelium in the mucosa or disability of cell division[32–37]. Secondly, the vascular and connective tissue changes can result in ischemia mucosa, which, when severe, can lead to mucosal ulceration and necrosis [33, 38]. When vascular injury is extensive, it can result in fibrosis, stenosis and reduction of normal intestinal motility,[39],which consequently increases the permeability of the mucosal cells, favoring the transfer of bacteria from the gastrointestinal tract to the mucosa [13,32, 40]. The growth of this bacteria is excessive, and disrupts the hosts’ immune defenses [17, 32, 41]. Subsequently, inflammation further accelerates the radiation response, the up-regulation of CD11/CD18 on leukocytes and NF-κB increase in the expression of endothelial cell adhesion molecules, which results in leukocyte adhesion to the vascular endothelium and subsequent extravasation of inflammatory cells into the inflamed tissue [42]. Moreover, inflammation can amplify endothelial dysfunction and increase the levels of cytokines and growth factors, such as transforming growth factor b (TGF-b), thus delaying the process of re-epithelialisation [34]. Radiation should also be responsible for the reduction of intestinal motility and bile acid reabsorption [14, 18, 43–45]. The last but not the least, radiation severely impaired intestinal micro villi, contributing to decrease enzymatic activity and total gut transit time [13, 46–53].

Probiotics are generally recognized as being an easy, safe and beneficial measures for the microbiota, thus it might be a feasible option approaching to effectively protect patients against the risk of radiation-induced diarrhea [54–56]. However, how do probiotics actually function? The potential associated benefits may list as follows. First, probiotics may alter the composition and metabolic activity of host’s microflora and therefore set a barrier by lowering intestinal pH [2, 32, 41, 57, 58]. Second, probiotics can enhance the mucosal barrier function and prevent bacteria from overgrowth by producing antibacterial substance [36, 59, 60]. Furthermore, probiotics may play a role in down-regulation of the intestinal inflammatory responses by triggering and regulating the function of immune cells, which favor recovery and homeostasis of intestinal mucosa [58–62]. Therefore, probiotics might be a promising pharmaceutical in preventing radiation-induced diarrhea.

### Implication for clinical practice

Radiotheraphy can cause severe and potential lethal complication for abdominal and pelvic cancers. Our meta-analysis broadly evaluated the available evidence which showed that probiotics could effectively reduce the incidence of the radiation-induced diarrhea and improve the quality of patients’ lives.

Accordingly, current evidence indicated that probiotics could be recommended as an adjunct for preventing radiation-induced diarrhea. Given the differences among the species and strains of probiotic, dosage, time and the set of studies and radiation, the urgent of clinical issue is how to use probiotics optimally. The main limitation of the primary outcome was influenced by various factors, which may affect the robustness of the conclusion and further confuse the clinical practice. Due to the difference of patients, treatment protocols, onset of intake as well as dosage, time, administration route and duration of delivery, species and strains of probiotics, it is difficult to compare our result with other studies [3, 13, 15, 17, 18, 41]. Consequently, based on current evidence, it may be uncertain to give a precise guidance on how to use probiotics to prevent the radiation-induced diarrhea. It should be a state-of-the-art technique to use probiotics regime to modulate the gastrointestinal microbiota appropriately and to further control the radiation-induced diarrhea.

### Strengths and limitations

Our meta-analysis included the latest and most convincing references which may be helpful for updates of the current guidelines. However, there are several limitations in our study. First, the dosage and the strains of probiotic are quite different in our study, there were four studies receiving *lactobacillus*(live *lactobacillus acidophilus*, *lactobacillus rhamnosus*, VSL no.3 and *lactobacillus casei* DN-114001) with the dosage ranging from 3×10^9^ CFU to 1.35×10^12^ CFU, and two studies receiving *lactobacillus acidophilus* plus *bifidobacterium bifidum with* the dosage ranging from 2.6×10^9^ CFU to 4 ×10^9^ CFU. Therefore, it may influence the primary outcome [63, 64]. Second, what is the most suitable time to take in would also be required further exploration [65]. It may be more effective to take in after lunch, because the food neutralized the gastric acid, which made it more smoothly to the intestinal. Moreover, the resultant variability among the extensive patient populations (Patient-related factors include smoking, body mass index, previous abdominal surgery and comorbidities), disease processes, disease complications and severity, treatment settings and drug resistance[65–69], which made it impossible to extend our finds to clinical applications, therefore, more well-designed treatment protocols remain to be settled. Third, therapy-related factors include radiation dose, volume of irradiated bowel, site of radiation (pelvic radiotherapy or in combination with intestinal radiatherapy), time and dose fractioning parameters, grading and staging of the tumor and concomitant employment of chemotherapy are different [69, 70]. Meanwhile, it is deserved to be noticed that the type of irradiation technique has been recognized as an influential factor, even the most recent radiation procedures. Intensity-modulated radiotherapy may not completely annulled the occurrence of radiation-induced diarrhea. Fourth, the criteria for diagnosis of diarrhea varied from study to study. Some studies evaluate the severity of diarrhea according to the National Cancer Institute Common Toxicity Criteria; NCI CTC version 2.0 (grade 0 = none;grade 1 = increase of < 4 stools/day over pre-treatment;grade 2 = increase of 4–6 stools/day, or nocturnal stools;grade 3 = increase of ≥ 7 stools/day or incontinence or need for parenteral support for dehydration; grade 4 = physiologic consequences requiring intensive care, or hemodynamic collapse) (17), while some studies evaluate the severity of diarrhea according to the World Health Organization (WHO): (grade 1 = increase of 2–3 stools per day compared to pre-treatment, grade 2 = increase of 4–6 stools per day or nocturnal stools, grade 3 = increase of 7–9 stools per day or incontinence, grade 4 = increase of 10 or more stools, IV hydration needed). Given this condition, it is essential to conduct a unified standard for diagnosis of diarrhea (13). Finally, and the most important, the number of included studies is only six. However, publication bias tests and plots are only relevant if having >10 studies included, otherwise, it is underpowered and tended to lead to conclusions that are not justified [71].

All factors mentioned above lead to a more meaningful effect on radiation-induced diarrhea prevention. Moreover, the results still could have been biased owing to the limitation of current studies. In addition, most of studies have not reported the adverse events of receiving probiotics treatment. Although, the incidence of side-effects may be low, it would also be well worth paying attention to.

## Conclusions

Our meta-analysis suggested that probiotics may have some beneficial effects on treatment of radiation-induced diarrhea in patients who suffered from abdominal or pelvic cancers during radiotherapy period. However, our study is only a selection of published studies, more well-designed, properly powered and randomized placebo-controlled trials are needed to reveal the real effectiveness of probiotic supplementation for the radiation-induced diarrhea.

## Supporting information

S1 Text PRISMA 2009 Checklist for this article. (DOC)

S2 Text Search strategy. (DOC)

## Abbreviations

RCTs: randomized controlled trials
PRISMA: Preferred Reporting Items for Systematic Reviews and Meta-Analyses
MeSH: Medical Subject Headings
RR: relative risk
CI: confidence interval
MD: mean difference
IV: Inverse variance
