# Supplementary material for: Probiotics for prevention of radiation-induced diarrhea: A meta-analysis of randomized controlled trials
Source: PLoS One. 2017 Jun 2;12(6):e0178870. doi: 10.1371/journal.pone.0178870 (PMC5456391; doi:10.1371/journal.pone.0178870)
Supplement: S2 Text — (DOC) [file pone.0178870.s002.doc]

**Appendix 1 Search strategy**

**Searched on: March 30, 2016**

**Searched source: PubMed**

**Searched results: 45**

| **PubMed** |  |  |
| --- | --- | --- |
| Search | Query | Results |
| #1 | "Diarrhea"[Mesh] | 46580 |
| #2 | Diarrhea[Title/Abstract] | 60105 |
| #3 | Diarrhoea[Title/Abstract] | 23288 |
| #4 | #1 OR #2 OR #3 | 97388 |
| #5 | "Radiotherapy"[Mesh] | 152628 |
| #6 | radiotherapy[Title/Abstract] | 142091 |
| #7 | radiation therapy[Title/Abstract] | 59477 |
| #8 | #5 OR #6 OR #7 | 262459 |
| #9 | #4 AND #8 | 1792 |
| #10 | Radiation-induced Diarrhea[Title/Abstract] | 52 |
| #11 | #9 OR #10 | 1804 |
| #12 | Probiotics[Title/Abstract] | 8585 |
| #13 | Synbiotics[Title/Abstract] | 422 |
| #14 | "Probiotics"[Mesh] | 10872 |
| #15 | "Synbiotics"[Mesh] | 218 |
| #16 | "Lactobacillus"[Mesh] | 23094 |
| #17 | Lactobacillus[Title/Abstract] | 21386 |
| #18 | Lactobacilli[Title/Abstract] | 6349 |
| #19 | "Bifidobacterium"[Mesh] | 4459 |
| #20 | Bifidobacterium[Title/Abstract] | 4933 |
| #21 | "Saccharomyces"[Mesh] | 97798 |
| #22 | Saccharomyces[Title/Abstract] | 64077 |
| #23 | Enterococci[Title/Abstract] | 9174 |
| #24 | bifidobacteria[Title/Abstract] | 3027 |
| #25 | 12 OR #13 OR #14 OR #15 OR #16 OR #17 OR #18 OR #19 OR #20 OR #21 OR #22 OR #23 OR #24 | 160690 |
| #26 | #11 AND #25 | 45 |

**Searched on: March 30, 2016**

**Searched source: Cochrane**

**Searched results: 14**

| **Cochrane** |  |  |
| --- | --- | --- |
| Search | Query | Results |
| #1 | Diarrhea:ti,ab,kw | 15760 |
| #2 | Diarrhoea:ti,ab,kw | 15760 |
| #3 | #1 or #2 | 15760 |
| #4 | radiotherapy:ti,ab,kw | 14851 |
| #5 | radiation therapy':ti,ab,kw | 7654 |
| #6 | #4 or #5 | 18126 |
| #7 | #3 and #6 | 692 |
| #8 | Radiation-induced Diarrhea':ti,ab,kw | 68 |
| #9 | #7 or #8 | 695 |
| #10 | Probiotics:ti,ab,kw | 2124 |
| #11 | Synbiotics:ti,ab,kw | 165 |
| #12 | Lactobacillus:ti,ab,kw | 2211 |
| #13 | Lactobacilli:ti,ab,kw | 589 |
| #14 | Bifidobacterium:ti,ab,kw | 1006 |
| #15 | Bifidobacteria:ti,ab,kw | 422 |
| #16 | Saccharomyces:ti,ab,kw | 273 |
| #17 | Enterococci:ti,ab,kw | 246 |
| #18 | #10 or #11 or #12 or #13 or #14 or #15 or #16 or #17 | 3948 |
| #19 | #9 and #18 | 18 |
| #20 | #9 and #18 in Trials | 14 |

**Searched on: March 30, 2016**

**Searched source: EMBASE**

**Searched results: 37**

| **EMBASE** |  |  |
| --- | --- | --- |
| Search | Query | Results |
| #1 | Diarrhea:ab,ti | 81882 |
| #2 | Diarrhoea:ab,ti | 29876 |
| #3 | #1 OR #2 | 110923 |
| #4 | radiotherapy:ab,ti | 184461 |
| #5 | radiation therapy':ab,ti | 85442 |
| #6 | #4 OR #5 | 250459 |
| #7 | #3 AND #6 | 2530 |
| #8 | Radiation-induced Diarrhea':ab,ti | 55 |
| #9 | #7 OR #8 | 2552 |
| #10 | Probiotics:ab,ti | 10934 |
| #11 | Synbiotics:ab,ti | 532 |
| #12 | Lactobacillus:ab,ti | 23652 |
| #13 | Lactobacilli:ab,ti | 7057 |
| #14 | Bifidobacterium:ab,ti | 6039 |
| #15 | Bifidobacteria:ab,ti | 3613 |
| #16 | Saccharomyces:ab,ti | 67197 |
| #17 | Enterococci:ab,ti | 10749 |
| #18 | #10 OR #11 OR #12 OR #13 OR #14 OR #15 OR #16 OR #17 | 114186 |
| #19 | #9 AND #18 | 37 |
